# Next-Generation Internet and Communication

**DOI:** 10.1155/2014/342471

**Published:** 2014-06-03

**Authors:** Weifeng Sun, Guoqiang Zhang, Jingjing Zhou, Vijay Bhuse

**Affiliations:** ^1^School of Software, Dalian University of Technology, Dalian 116620, China; ^2^Nanjing Normal University, Jiangsu 210023, China; ^3^School of Information and Electronic Engineering, Zhejiang Gongshang University, Zhejiang 310018, China; ^4^Department of Computing, East Tennessee State University, Johnson City, TN 37614-1266, USA

The Internet is one of the most key technical infrastructures in existence today. It will be a catalyst for much of our innovation and prosperity in the future. However, the present Internet may not be capable of meeting so many requests much longer. Fortunately, scientists and engineers believe that new technologies, protocols, and standards can be developed to meet tomorrow's demands. The research on this new Internet is considered as a global research challenge. Therefore, we have organized a special issue for researchers, engineers, and practitioners to share their work on next-generation Internet technologies.

Nowadays, the researches about next-generation Internet mostly focus on the design, engineering, protocols, and operation the of the new signal processing techniques in 3G/4G/B4G/LTE [1, 2], Ad Hoc Network and Wireless Mesh Network [3, 4], cloud computing [5], Internet of things (IoT) [6], channel allocation [7], and some other fields.

The papers in this special issue of next-generation Internet and communication cover a wide range of topics from underlying infrastructure and protocols to high-level systems design and analysis.

The new signal processing techniques in 3G/4G/B4G/LTE are a hot issue, and for this topic, we accepted three papers. They talk about the channel allocation and transmission problems in LTE.

Ad Hoc Network/Wireless Mesh Network has been a hot topic for several years and it is still a hot topic. The Wireless Mesh Network is Ad Hoc Network essentially, so we regard them as one topic. We accepted seven papers for this topic. They focus on the routing protocols and data delivery method in Ad Hoc Network or Wireless Mesh Network and propose some new routing protocols, such as a source-initiated on-demand routing algorithm.

Another important topic is channel allocation. We accepted six papers for this topic, including a paper about routing scheme. They talk about how to gain the network transfer capacity.

Security is another basic issue. For this topic, we accepted two papers. One is about defense model based on fuzzy evaluation. In the paper, first, the authors propose a distributed network security architecture, comprising a hybrid firewall, intrusion detection, virtual honey net projects, and connectivity and interactivity between these three components. The other one is about multiple-feature extracting modules. In the paper, they implement a crawler mining system that is equipped with SQL injection vulnerability detection, by means of an algorithm developed for the web crawler.

Cloud computing and IoT are very hot in recent years. We accepted two papers. One is about cloud computing. In the paper, the authors propose a novel unfair rating filtering method for a reputation revision system using prior knowledge as the basis of similarity when calculating the average rating, which facilitates the recognition and filtering of unfair ratings. The other one is about IOT. IOT accumulates large amounts of data by IOT users, which is a great challenge to mining useful knowledge from IOT. Classification is an effective strategy which can predict the need of users in IOT. In the paper, a new rule-based classification is proposed.

All the researches about Internet are for the purpose of getting better services, so the services of Internet are also important topics. For this topic, we accepted three papers. They are about social network, content centric Internet, and power WebGIS platform.

We hope that readers will find in this special issue not only the new ideas, cutting-edge information, new technologies, and applications of next-generation Internet and communication, but also a special emphasis on how to solve various problems.

*Weifeng  Sun* *Weifeng  Sun* *Guoqiang  Zhang* *Guoqiang  Zhang* *Jingjing  Zhou* *Jingjing  Zhou* *Vijay  Bhuse* *Vijay  Bhuse*
